# Tumor infiltrating B-cells are increased in prostate cancer tissue

**DOI:** 10.1186/1479-5876-12-30

**Published:** 2014-01-30

**Authors:** Jason R Woo, Michael A Liss, Michelle T Muldong, Kerrin Palazzi, Amy Strasner, Massimo Ammirante, Nissi Varki, Ahmed Shabaik, Stephen Howell, Christopher J Kane, Michael Karin, Christina AM Jamieson

**Affiliations:** 1 Department of Urology and Department of Surgery, University of California San Diego, La Jolla, CA, USA; 2 Department of Pathology, University of California San Diego, La Jolla, CA, USA; 3 Department of Pharmacology, University of California San Diego, La Jolla, CA, USA; 4 Department of Medicine, University of California San Diego, La Jolla, CA, USA; 5 Moores Cancer Center, University of California San Diego, La Jolla, CA, USA; 6 Department of Urology, UCSD Moores Cancer Center, 3855 Health Sciences Drive, Mail Code: 0987, La Jolla, CA 93093-0987, USA

**Keywords:** CRPC: castrate-resistant prostate cancer, TIL: tumor infiltrating lymphocyte, B-cells, CD20, Immunohistochemistry, Prostatectomy, D’Amico risk stratification, Digital IHC image analysis

## Abstract

**Background:**

The presence of increased B-cell tumor infiltrating lymphocytes (TILs) was seen in mouse prostate cancer (PCa) but has not been fully documented in human PCa. We, therefore, investigated the density of infiltrating B cells within human PCa utilizing a quantitative computational method.

**Methods:**

Archived radical prostatectomy specimens from 53 patients with known clinical outcome and D’Amico risk category were obtained and immunohistochemically (IHC) stained for the B cell marker, CD20. Slides were reviewed by a genitourinary pathologist who manually delineated the tumoral regions of PCa. Slides were digitally scanned and a computer algorithm quantified the area of CD20 stained B-cells as a measure of B cell density within the outlined regions of prostate cancer (intra-tumoral region), versus extra-tumoral prostate tissue. Correlations were analyzed between B-cell density and demographic and clinical variables, including D’Amico risk groups and disease recurrence.

**Results:**

For the entire cohort, the mean intra-tumoral B cell density was higher (3.22 SE = 0.29) than in the extra-tumoral region of each prostatectomy section (2.24, SE = 0.19) (paired t test; P < 0.001). When analyzed according to D’Amico risk group, the intra-tumoral B cell infiltration in low risk (0.0377 vs. 0.0246; p = 0.151) and intermediate risk (0.0260 vs. 0.0214; p = 0.579) patient prostatectomy specimens did not show significantly more B-cells within the PCa tumor. However, patient specimens from the high-risk group (0.0301 vs. 0.0197; p < 0.001) and from those who eventually had PCa recurrence or progression (0.0343 vs. 0.0246; p = 0.019) did show significantly more intra-tumoral CD20+ B-cell staining. Extent of B-cell infiltration in the prostatectomy specimens did not correlate with any other clinical parameters.

**Conclusions:**

Our study shows that higher B-cell infiltration was present within the intra-tumoral PCa regions compared to the extra-tumoral benign prostate tissue regions in prostatectomy sections. For this study we developed a new method to measure B-cells using computer-assisted digitized image analysis. Accurate, consistent quantitation of B-cells in prostatectomy specimens is essential for future clinical trials evaluating the effect of B cell ablating antibodies. The interaction of B-cells and PCa may serve as the basis for new therapeutic targets.

## Background

Understanding of the immune system’s role in modulation of solid malignancies has increased significantly in recent years. Both the innate and adaptive branches of the host immune system have been shown to mount responses against prostate tumor cells [1,2]. Immunotherapeutics represent a new paradigm in treating PCa by augmenting the host immune system [1]. Current immunotherapeutics in use or under investigation include tumor vaccines that enhance the function of antigen presenting cells (APCs) and monoclonal antibody immunotherapeutics known as checkpoint modulators that are now the target of several therapeutics in the clinic [3]. All these drugs ultimately target T-cell mediated responses [3]. However, the role of B-cell immunity in PCa is not well understood, and represents an opportunity for further investigation and therapeutic development.

Recently, CD20^+^ B-cell TILs were found to have prognostic significance in melanoma, breast, non-small cell lung cancer, and ovarian cancers [3-6]. However, the presence of increased B-cell TILs has not been systematically investigated in PCa [7-9]. Pre-clinical models have demonstrated that B-cells play an important role in castrate-resistant PCa (CRPC) [10-12]. Androgen ablation in murine models and the consequent PCa cell death cause damage to the stromal cell compartment of the tumor microenvironment, that elicit leukocyte recruitment, including B cells, that promote PCa recurrence [10,11]. B cell recruitment depends on the chemokine, CXCL13, whose expression is induced in response to androgen ablation [11]. Importantly, the tumor infiltrating B cells produce lymphotoxin (LT), a heterotrimeric cytokine that belongs to the TNF family, leading to activation of IκB kinase α (IKKα) and STAT3 which promote survival and proliferation of androgen-deprived PCa cells resulting in development of CRPC [11]. IKKα activation results in phosphorylation of E2F1 and enhanced transcription of the BMI1 gene which encodes a component of the polycomb ubiquitin ligase complex [11,12]. Elevated expression of BMI1 results in accumulation of ubiquitinated histones in PCa nuclei [10-12]. These molecular events are required for B cell-dependent, castrate-resistant regrowth of the prostate cancer cells [11].

As a first step towards translation of the murine studies to the clinic, we investigated the density of B-cells within PCa tumor tissue from patients with differing risks of PCa recurrence. The presence and location of B-cells within prostate cancer tissue from patients who underwent prostatectomy was investigated utilizing a reproducible and quantitative computational method of identifying B-cells.

## Methods

### Selection of cases and patient population

After approval from the UCSD institutional review board (IRB), 53 formalin-fixed, paraffin-embedded (FFPE) radical prostatectomy specimens from separate patients were subjected to CD20 immunohistochemical (IHC) analysis. The patients were selected based on known clinical outcome according to risk categories of low, intermediate and high-risk groups based on the D’Amico risk classification [13], as well as a group of patients with disease recurrence. Additional clinical characteristics of each patient including age, race, body mass index (BMI), co-morbidities, family history, preoperative PSA, Gleason score, and pathological data were obtained.

### Immunohistochemistry

Radical prostatectomy specimens were cut into 5 mm axial sections, formalin-fixed and routinely processed for paraffin embedding. Subsequently the embedded specimens were cut into 5 μm sections and stained with hematoxylin and eosin (H and E). Paraffin sections were deparaffinzed, rehydrated, and blocked for endogenous peroxidases and endogenous biotin. Epitopes were revealed using antigen retrieval in citrate buffer, pH 6.0 (DAKO catalog # S1700). Slides were incubated with mouse anti-CD20 antibody (DAKO, catalog # MO755) in blocking solution consisting of 1% BSA, PBS-Tween 20. Bound anti-CD20 antibody was detected using the Envision Horseradish peroxidase (HRP)-labeled anti-mouse secondary antibody (DAKO catalog # K4000) and 3-amino-9-ethylcarbazole (AEC) substrate chromagen (DAKO catalog # 3464) then counter-stained with hematoxylin [14].

### Data collection and image analysis

**Figure 1:**
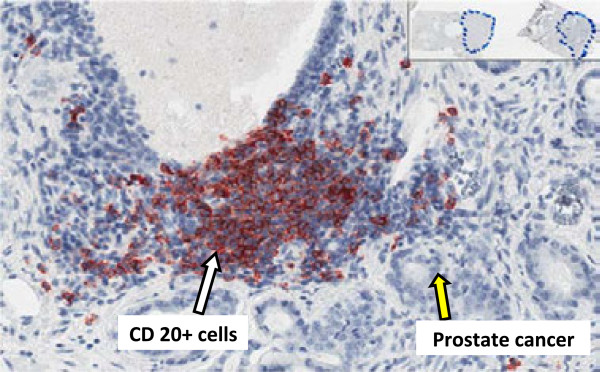
**Immunohistochemical staining of prostatectomy sections with anti-CD20 antibody.** Paraffin-embedded, formalin fixed prostatectomy sections were stained with anti-CD20 and counterstained with hematoxylin, The majority of CD20 immunostained B-cells are localized in immune cell foci. Many in close proximity to prostate cancer cells as shown. Inset shows whole slide scan with blue-pen marked tumor regions in each prostatectomy section.

A board-certified genitourinary pathologist (A.S.) reviewed IHC-stained slides, and the areas of tumor were manually identified and marked on the slides (Figure 1a and b). All slides were scanned using the Aperio ScanScope XT system (Aperio®; Vista, CA). The Spectrum Analysis algorithm package and ImageScope analysis software were applied to quantify IHC staining. The raw image data were in RGB format in which the colors of red, blue, and green make up a 256-level scale. Colors were represented as discrete variables, and each color was assigned a numerical value between 0 and 255. The color derived at each pixel was represented digitally by its three separate values indicating the level of red, green, and blue. The “color deconvolution” algorithm (version 9, Aperio®, Vista, CA) was created to quantitatively analyze the slides for the presence of the AEC stain corresponding to CD20+ B-cells. This algorithm identifies the AEC stained areas, based on specific thresholds for the presence of red, green and blue established for AEC. Areas of tissue folds, pen marks, and air bubbles were identified and excluded from our analysis as they disrupted the software analysis.

### Data analysis

The primary outcome was the mean density of B-cells within the area of tumor versus the B-cell density outside of the tumor area. B-cell density was recorded for the entire specimen, within PCa tumor region (intra-tumoral), and outside tumor region (extra-tumoral) using the following calculations:

$$\begin{array}{c} \frac{\mathrm{AEC}\;\mathrm{positive}\;\mathrm{area}\;\mathrm{intra}-\mathrm{tumoral}\;\mathrm{region}\left(mm^{2}\right)}{\mathrm{Total}\;\mathrm{Intra}-\mathrm{Tumoral}\;\mathrm{area}\;\left(mm^{2}\right)}\;\mathrm{and}\;\\ \frac{\mathrm{AEC}\;\mathrm{positive}\;\mathrm{area}\;\mathrm{within}\;\mathrm{prostate}\;\mathrm{tissue}\;\left(mm^{2}\right)}{\mathrm{Total}\;\mathrm{Extra}-\mathrm{tumoral}\;\mathrm{prostate}\;\mathrm{area}\;\left(mm^{2}\right)} \end{array}$$

Samples were analyzed based on the three D’Amico Risk groups and in a population of patients who had recurrence in a pilot analysis. B-cell density was skewed to the right; therefore, the non-parametric Kruskal-Wallace test was used to compare the overall B-cell density in each D’Amico risk group or Gleason group (<6, 7, and >7). Paired-t test was utilized to determine the location of the B-cells within each prostatectomy specimen and graphically displayed in a difference plot. The statistics package SPSS v.21 (IBM, Chicago, IL, USA) was used.

## Results

The aim of this study was to determine the potential of using B-cell infiltration as a prognostic marker of PCa recurrence. CD20 IHC was performed on a cohort of 53 paraffin-embedded radical prostatectomy specimens from D’Amico risk-stratified patients selected from the UCSD Urologic Oncology database. Demographics and clinicopathological features for the cohort are displayed in Tables 1 and 2. Diagnoses potentially related to inflammation such as hypertension, hypercholesterolemia, and coronary artery disease remained relatively low in the population. B-cell density was measured as the area of CD20 positive B-cells (mm^2^) per analyzed area of prostatectomy section (mm^2^). The analyzed areas were: the total B-cell density of the entire area of the specimen, the intra-tumoral B-cell density within the outlined PCa tumor area, and extra-tumoral B-cell density outside the tumor area. The primary outcome of this study was to determine the location of highest B-cell density in prostatectomy specimens.

**Table 1:** Demographic and clinical characteristics

	***ALL (n = 53)***
Mean age ± SD, years	62 ± 6.8
Race	
Caucasian	45 (84.9%)
Other	8 (15.1%)
Mean BMI ± SD, Kg/m2	27.7 ± 3.6
Hypertension	13 (24.5%)
Hypercholesterolemia	15 (28.3%)
Diabetes	5 (9.4%)
Coronary artery disease	4 (7.5%)
5 Alpha-Reductase (Proscar/Avodart)	8 (15.1%)
Primary relative with prostate Ca	17 (32.1%)
Median PSA (IQR), ng/mL	6.2 (4.2–9.8)
Clinical T stage	
T1a-c	24 (45.3%)
T2a-c	25 (47.2%)
T3a-b	4 (7.5%)
Biopsy Gleason score	
≤ 6	20 (37.7%)
7	17 (32.1%)
≥ 8	16 (30.2%)
D'Amico risk group	
Low risk	10 (16.7%)
Intermediate risk	9 (15.0%)
High risk	15 (25%)
Recurrence	26 (43.3%)

**Table 2:** Operative and pathological details and outcomes

	***ALL (n = 53)***
Median operative time (IQR), mins	189 (170–200)
Median estimated blood loss (IQR), mL	150 (100–200)
Median prostate weight (IQR), gm	44.7 (38–57.1)
Blood transfusions	1 (1.8%)
Lymph node dissection	37 (69.8%)
Complete nerve sparing	37 (69.8%)
Median hospital stay (IQR), days	1 (1–1)
Median lymph nodes retrieved (IQR)	17 (12–21)
Positive LN	5 (9.4%)
Median tumor size (IQR), cc	6.9 (2.6–13.7)
Positive margins	20 (41.5%)
Seminal vesicle involvement	6 (11.3%)
Extra capsular extension	15 (28.3%)
Perineural invasion	44 (83.0%)
Extensive prostatic intraepithelial neoplasia	5 (9.4%)
Vascular/Lymphatic invasion	10 (18.8%)
Pathologic T stage	
T2a-c	29 (54.7%)
T3a-b	21 (39.6%)
T4	3 (5.7%)
Pathologic Gleason score	
≤ 6	13 (24.5%)
7	16 (30.2%)
≥ 8	24 (45.3%)

**Figure 2:**
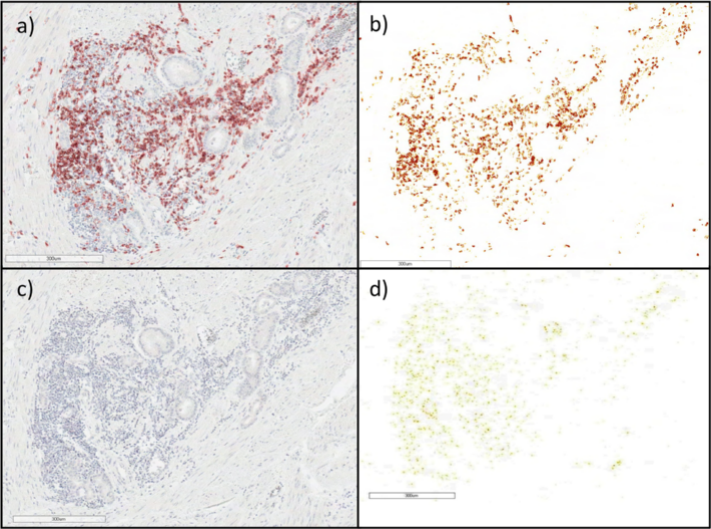
**Computer-supported digital quantification of analysis CD20 immunohistochemical staining of prostatectomy sections. a** prostatectomy section anti-CD20 stained slide, **b** serial section stained with isotype negative control antibody (mouse IgG). **c** Imagescope deconvolution algorithm is applied, color intensity is converted to representation as image pixels with high intensity (brown), intermediate (orange) and low (yellow) staining intensity. **d** represents the isotype negative control antibody (mouse IgG) in **b** after applying the deconvolution algorithm.

**Figure 3:**
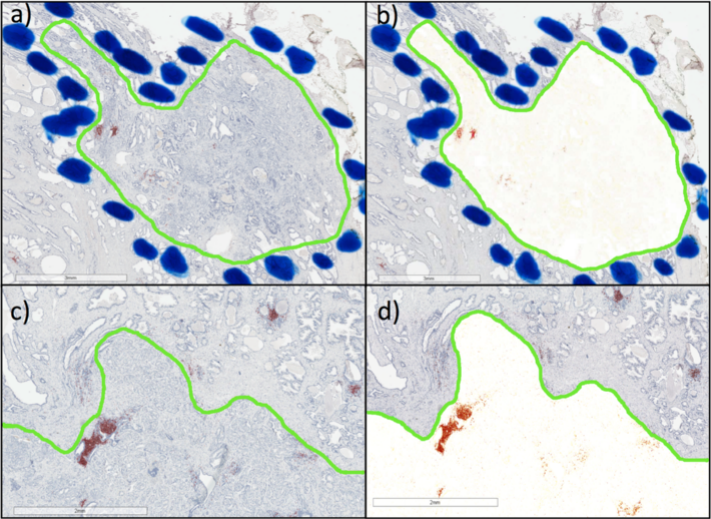
**Tumor region selection and digital quantification.** A genitourinary pathologist manually marked the tumor regions in each section on each slide using blue permanent pen marks **(a and b)**. Using ImageScope software the tumor regions were digitally outlined as shown with the solid green lines. The deconvolution algorithm was applied to the outlined tumor region as shown in **c** and **d**. The intra-tumoral B cell density was quantified as the area of CD20 stained (mm^2^) of AEC-positive cells **(c and d)**.

The majority of anti-CD20 immunostained B-cells were localized in immune cell foci, and many B-cells were in close proximity to prostate cancer cells (Figure 1). Specificity of immunostaining was shown using the anti-CD20 antibody stained specimen (Figure 2a) compared to the isotype negative control antibody as shown in (Figure 2c). Slides were digitally scanned and pixel staining intensity was quantified using an AperioScope deconvolution algorithm (described below) in which staining intensity pixels were represented as pseudo colors: high intensity as red, intermediate intensity as orange and low intensity as yellow (Figure 2b). Serial sections stained with isotype control antibody were used to set threshold of non-specific background staining (Figure 2d). The area of tumor within each slide was then outlined digitally using the ImageScope software (Figure 3). Within each area of tumor, the total area (mm^2^) of AEC-positive cells was calculated using the color deconvolution algorithm as described above (Figure 3b and d). This was then repeated for the area of AEC-positive cells within the non-tumor areas of the specimen.

The primary hypothesis was that there would be more B-cells within the tumor than surrounding non-tumor areas. Using paired statistical analysis to account for individual variation, the mean intra-tumoral B-cell density was higher (3.22 SE = 0.29) than outside the tumor region (extra-tumoral) (2.24, SE = 0.19) (paired t-test; P < 0.001) in the whole cohort.

Correlations between B-cell density and demographic variables were investigated, with attention to inflammatory mediated disease such as hypertension, hypercholesterolemia, and coronary artery disease. No correlations were noted between overall B-cell density or intra-tumoral B cell density compared to age, body mass index, race, hypertension, hypercholesterolemia, diabetes, family history of prostate cancer, coronary artery disease or history of 5-alpha reductase inhibitors (all p > 0.05).

**Figure 4:**
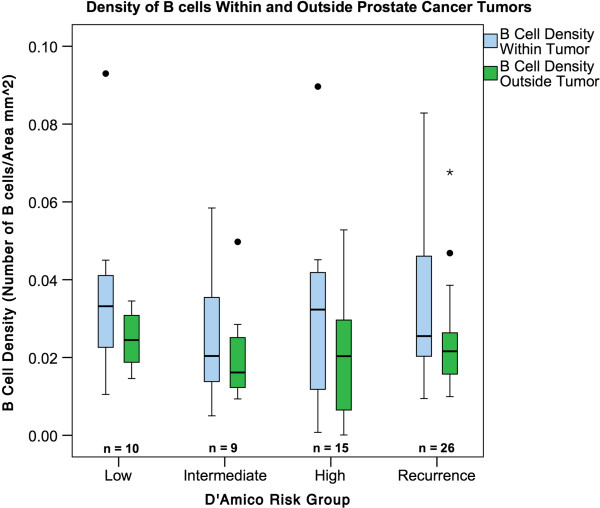
**Intra-tumoral B-cell density compared to and extra-tumoral B cell density in each D'Amico risk group.** Box plots representing the B-cell density inside the delineated tumor region (intra-tumoral) and outside the tumor regions (extra-tumoral) in radical prostatectomy speciments. B cell density was defined as: the area of CD20 positive staining (mm^2^)/ total area analyzed ie. intra-or extra-tumoral prostatectomy tissue region in each section. The B cell densities in the intra-tumoral regions were compared to the extra-tumoral regions in each of the D'Amico risk groups: low (n = 10), intermediate (n = 9), and high (n = 15) and in the group of patients with known prostate cancer recurrence (n = 26). The median B cell density is shown as the horizontal line in each box plot. Asterisk over recurrence group indicates outlier.

Samples were analyzed based on the three D’Amico Risk groups and in a population of patients who had recurrence in a pilot analysis. B-cell density was skewed to the right; therefore, the non-parametric Kruskal-Wallace test was used to compare the overall B-cell density in each D’Amico risk group or Gleason group (<6, 7, and >7). Comparing the B-cell density within the tumor and outside the tumor in D’Amico low risk (0.0377 vs. 0.0246; p = 0.151) and intermediate risk (0.0260 vs. 0.0214; p = 0.579) did not show significant differences (Figure 4). However, the D’Amico high-risk group and recurrence group did show more intra-tumoral CD20+ B cell staining compared to extra-tumoral (0.0301 vs. 0.0197; p < 0.001) and (0.0343 vs. 0.0246; p = 0.019), respectively (Figure 4).

**Figure 5:**
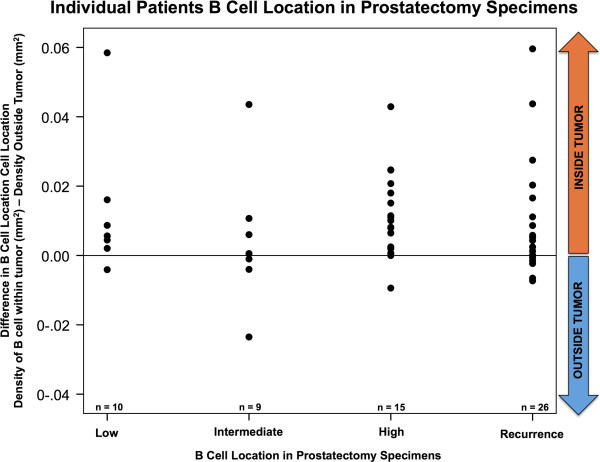
**Individual patients B-cell location in prostatectomy specimens.** The B-cell densities (mm^2^) were measured after CD20 staining of radical prostatectomy and computer generated values of area of staining was divided by area analyzed. This density was calculated within the tumor and outside the tumor and the difference was compared using the paired t-test. Each dot represents an individual patient’s intra-tumoral B-cell density minus their extra-tumoral B-cell density to display the magnitude of difference in B-cell location per patient.

In order to take into account the variation between patients in their overall B cell density in the prostate, the B cell densities for individual patients were determined based on their location in the prostatectomy specimens, that is, intra-tumoral compared to extra-tumoral regions. The B-cell densities (mm^2^) were measured after CD20 staining of radical prostatectomy and computer generated values of area of staining was divided by area analyzed. This density was calculated within the tumor and outside the tumor and the difference was compared using the paired t-test. As shown in Figure 5 each dot represents an individual patient’s intra-tumoral B-cell density minus their extra-tumoral B-cell density to display the magnitude of difference in B-cell location per patient. For the entire cohort, the mean intra-tumoral B cell density was higher (3.22 SE = 0.29) than in the extra-tumoral region of each prostatectomy section (2.24, SE = 0.19) (paired t test; P < 0.001).

## Discussion

The results of our study show that intra-tumoral B-cells were present in higher numbers in comparison to the extra-tumoral regions of adjacent non-malignant tissue in each of the radical prostatectomy specimens. To reduce sampling error and intra- and inter-observer variability, B-cells were quantitated using computer supported image analysis.

Tumor infiltrating lymphocytes (TILs), particularly CD8+ T cells, have been shown to have strong positive prognostic relevance in multiple other solid tumors [15-17]. However, the role of B-cell immunity in PCa is not well understood, and represents an opportunity for study and potential therapy. The majority of studies have focused on CD8+ T cells, which have been recognized as the lymphocytes with the strongest anti-tumor activity [15-17]. Previously, B-cells have been reported as less abundant in prostate tissue than T-cells or macrophages [15-18]. However, analyses of B-cell density have varied, depending on tissue type, histological methods, and technique used. Hussein *et al.* reviewed 50 transurethral resection of prostate specimens with high-grade PCa, and found that the number of manually-counted CD20+ cells was significantly higher in adenocarcinoma than in normal prostate [19]. Fujii *et al.* reviewed 100 radical prostatectomy specimens by manually estimating lymphocyte percentages and showed opposing results, as the frequency on B-cells in benign tissue was actually higher in benign tissue than in adenocarcinoma [8]. Other investigators used tissue microarrays (TMA) to quantify B-cells [18-21]. A limitation of TMAs in quantifying prostatic B-cells is sampling error, as prostate adenocarcinomas are often heterogeneous and larger than the tissue cylinder used in TMA. B-cells in particular are scant and variable, often forming into sporadic clusters that can increase sampling error when using such methods [20-23]. Our computer supported analysis of the whole prostatectomy section as well as specific regions within each section allowed the quantification of large amounts of tissue to confirm a statistically significant higher number of B-cells in the tumor regions of each tissue section. While TMA analyses sample an approximately 0.28 mm^2^ area of tissue, our area of analysis was approximately 300 mm^2^ per sample. This is a novel implementation of the computer image supported analysis in prostate tissue.

To overcome the limitations of sampling error and intra- and inter-observer variability in quantifying lymphocytes, computer-supported image analysis was used to quantify the area of the CD20^+^ B-cells over the entirety of the radical prostatectomy specimens. Protein quantification by IHC remains semi-quantitative and is subject to variability between interpreters with the use of manual (‘eyeballing’) scoring systems [24]. Digital quantification of IHC staining was first described 20 years ago, but until recently its adoption has been limited due to insufficient technology and lack of validated standards in image acquisition and analysis [25-27]. CD20^+^ staining is specific to B-cells and the color of the AEC stain is distinct from any background staining, so color analysis rather than morphological analysis is accurate. The images are saved in RGB format with each color represented as a variable on the 256-color scale, thus we are able to establish discrete color thresholds that will allow the software to identify the specific color of the AEC stain. Given the range of possible color thresholds, this allows for analysis of a continuous spectrum of results rather than a pre-defined visual scoring system (e.g. “present vs absent” or “0, 1, 2, 3”). This allows for the more flexible statistical evaluation such as multi-parametric calculations, which may reveal associations not present in manual analyses [27]. Given the reliability of this technique in our experience, we anticipate future use of digital image assessment in prostate B-cell research and, perhaps, in the clinical setting.

Analysis of B cell density in these prostatectomy specimens failed to demonstrate a correlation between the intra-tumoral density of B-cells and the separate clinical and pathological parameters from our patient cohort. This is consistent with prior series that did not find associations between B-cell TILs and patient clinical features [18,21]. Prior studies with TMA analyses of radical prostatectomy specimens showed that B-cells were not associated with PSA-free survival, clinical stage, lymph node status, Gleason score or PSA [18,21]. The prognostic implications of T-cells in PCa tissue also remains controversial, with conflicting evidence linking high T-cell counts to both negative and positive prognosis [18,21,28-30]. In other better-studied solid malignancies such as colorectal and ovarian cancer, higher intra-tumoral T-cell counts have been associated with better clinical outcomes, indicating the biological impact of the immune system on tumor development and progression [21].

Immunotherapies for PCa treatment have targeted T-cell pathways. Sipuleucel-T (PROVENGE®; Dendreon, Seattle, WA) is a tumor vaccine that enhances the function of antigen presenting cells and T-cells, and is FDA approved for asymptomatic or minimally symptomatic CRPC [31]. Ipilimumab (YERVOY™; Bristol-Myers Squibb, Princeton, NJ) is a monoclonal antibody against cytotoxic T-lymphocyte antigen 4 (CTLA-4), which is an immune checkpoint regulator [3,32]. Immune checkpoint blockade “takes the brakes off the immune system” and improves anti-tumor responses. Occupancy of CTLA-4 dampens T-cell response against PCa [32]. Ipilimumab is currently in two Phase III clinical trials in patients with metastatic castrate resistant prostate cancer (mCRPC): http://1.usa.gov/ZKqSlg and http://1.usa.gov/146x4Hv#sthash. eS72VER7.dpuf.

Monoclonal antibodies against another checkpoint modulator, PD-1, such as nivolumab (Bristol-Myers-Squibb, BMS-936558), are also showing preliminary efficacy in clinical trials for a number of cancers, though no objective responses have been observed yet in Phase 1 trials in CRPC patients [33]. PD1 is thought to regulate T cell activation at a later stage than CTLA4 and is expressed on a wider range of immune cells including activated T cells in the periphery, regulatory T cells known as Tregs, activated B cells and NK cells [3,32]. Its ligands PD-L1 and PD-L2 are expressed on immune cells and non-immune cells including on some tumors. Inhibition of PD1 signaling by anti-PD-L1 antibody therapeutics are currently in clinical trials for a range of cancers including prostate cancer [3,32,33].

Humoral immunity is of particular interest in PCa as chronic inflammation appears to play a role in tumorigenesis and metastatic progression, with emerging literature supporting a role for B-cell derived cytokines in PCa progression [16,30]. After androgen ablation, B cell infiltration into prostate tumor results in increased production of LT and activation of IKKα and STAT3 to promote the emergence of CRPC [11]. Our analysis of B cells in prostate tumors in this study lays the groundwork for future clinical trials in which we plan to use this method to quantify B cells along with other pathologic data or staining of additional markers such as CXCL13, IKKα and BMI1 that were identified in the murine preclinical studies into more quantitative analysis in patients [11,12]. We used this study of B-cell quantitation as a recent example for an upcoming clinical trial which we powered by using the paired T-test with post-operative prostatectomy specimens in treated and untreated patients using this technique with a sample size of twenty in each group.

The results must be viewed within the limitations of the study and analysis of additional patients is necessary to verify the results. The study did not show any association between the number of B-cells and patient clinical parameters, and the study may be underpowered to detect differences between the risk groups, especially given the low numbers of CD20^+^ cells in each specimen. However, the primary hypothesis that B-cells were higher in malignant tissue (intra-tumoral) versus benign tissue (extra-tumoral) was supported by the results.

## Conclusions

The findings of this study have implications for immunotherapy to treat prostate cancer. The first step in recognizing the B-cell pathway as a potential target in prostate cancer is to consistently document the presence of CD20+ cells in malignant tissue, which we have done in a reproducible and quantitative computational method.

## Abbreviations

AEC: 3-amino-9-ethylcarbazole; APCs: Antigen presenting cells; BMI1: BMI1 polycomb ring finger oncogene; CTLA-4: Cytotoxic T-lymphocyte antigen 4; CXCL13: Chemokine (C-X-C motif) ligand 13 (ANGIE, ANGIE2, BCA-1, BCA1, BLC, E2F1); E2F: Transcription factor 1; FFPE: Formalin-fixed, paraffin-embedded; hematoxylin and eosin (H and E); Horseradish peroxidase (HRP); IHC: Immunohistochemical; IKKα: IκB kinase α; PD-1: Programmed cell death 1; PD-L1: Programmed cell death ligand 1, CD274 molecule (B7-H, B7H1); PD-L2: Programmed cell death 1 ligand 2 (BLR1L, SCYB13); PCa: Prostate cancer; PSA: Prostate specific antigen, kallikrein-related peptidase 3; STAT3: Signal transducer and activator of transcription 3 (acute-phase response factor); TMA: Tissue microarray.

## Competing interests

The authors declare that they have no competing interests.

## Authors' contributions

JRW, was involved in development, design and testing of digital scanning methods and analysis strategies, performed ImageScope analysis of outlined tumor regions on digitized images, compared analysis algorithms then applied final deconvolution algorithm, collected data and created data table of CD20 IHC quantitation on all patient sections. Prepared Tables 1 and 2, Figures 2 and 3. Wrote first draft and edited manuscript. MAL, designed and performed correlational and statistical analyses of B cell density in whole cohort, D’Amico risk stratified groups, clinical characteristics, and the difference in B cell location in each patient section that revealed higher intra-tumoral B-cell density. Conceived of data comparisons and created Figures 4 and 5. Wrote and edited manuscript. MTM, was involved in performing digital image analysis of outlined tumor regions with ImageScope and applied deconvolution algorithm. Contributed to final data table of CD20 IHC quantitation. Involved in selecting and collecting digital images for Figures 1, 2 and 3. KP, managed Urologic Oncology Database, created patient and clinical information database of selected cohort, helped with IRB protocol and statistical analysis. AS, helped with experiment design, IHC methodology and digital analysis. Involved in writing and editing manuscript. MA, helped with experiment design and IHC methodology, performed preliminary IHC analysis on TMA. NV, directed performance of CD20 IHC on all patient sections, comparison of IHC methods with lowest background required for accurate digital CD20 IHC quantitation, advised on digital scanning method and analysis. Involved in writing and editing manuscript. AS was the clinical pathologist who reviewed of all hematoxylin and eosin stained slides of full cohort of 53 prostatectomy cases and selected best paraffin blocks that contained tumor tissue and best representative slides for each patient. Evaluated all CD20 stained slides and estimated % CD20^+^ B cells in inflammatory infiltrates in sections. Manually outlined tumor regions on all prostatectomy sections. Advised on quantitative IHC analysis methods. Involved in writing and editing manuscript. SH, guided and advised effort to focus on developing a clinically informative and quantitative measurement of B-cells in prostate tissue. Involved in writing and editing manuscript. CJK, designed and selected cohort of patients to study based on D’Amico risk stratification and selected patients from the UCSD Urologic Oncology database. Devised formulae for calculations of B-cell density as a measure of area of CD20 staining in the intra-tumoral region compared to extra-tumoral or whole tissue section. Involved in writing and editing manuscript. MK, led research establishing that B cells promote CRPC in murine models and demonstrated an increase in B-cells in PCa of increasing malignancy in TMA analysis. This research was the rationale for performing this study on a larger, risk-stratified PCa patient cohort with known outcomes. Guided IHC method development and optimization. Involved in writing and editing manuscript. CAMJ, led and coordinated CD20 IHC analysis of the patient cohort. Conceived of the idea to measure B-cells next to the prostate tumor cells separately from B cells in adjacent benign prostate tissue. Formulated the hypothesis that intra-tumoral B-cells would be more numerous in PCa than in benign prostate tissue. Designed, developed, optimized and finalized quantitative CD20 IHC analysis strategy for quantitation of intra-tumoral B-cell and extra-tumoral B-cell density in consultation with co-authors. Performed CD20 IHC digital image analysis and applied deconvolution algorithm. Planned, wrote and edited manuscript, designed figures, wrote and edited manuscript. All authors read and approved the final manuscript.
